# Use of AI within COA linguistic validation and eCOA migration processes: analysis and good practice recommendations

**DOI:** 10.1186/s41687-026-01012-5

**Published:** 2026-02-06

**Authors:** Shawn McKown, Benjamin Arnold, Frederique Boucher, Helena Correia, Sonya Eremenco, Tomislav Geršić, Melinda Johnson, Andriana Koumbarou, Igor Matias, Emily Parks-Vernizzi, Tim Poepsel, Jiyoung Son, Erin Strouse, C. B. Terwee, Mark Wade

**Affiliations:** 1 IQVIA, Durham, NC USA; 2 FACITtrans, Ponte Vedra, FL USA; 3 Mapi Research Trust, Lyon, France; 4 Department of Medical Social Sciences, Northwestern University Feinberg School of Medicine, Chicago, IL USA; 5 Critical Path Institute, Tucson, AZ USA; 6 Borliner, Karlovac, Croatia; 7 RWS, East Hartford, CT USA; 8 Health Psychology Research Ltd., Egham, UK; 9 Quality of Life Technologies Lab, University of Geneva, Carouge, Switzerland; 10 Cognitive Aging Lab, University of Geneva, Carouge, Switzerland; 11 Eli Lilly and Company, Indianapolis, IN USA; 12 Amsterdam UMC location Vrije Universiteit, Department of Epidemiology and Data Science, Amsterdam, the Netherlands; 13 Amsterdam Public Health Research Institute, Methodology, Amsterdam, the Netherlands; 14 TransPerfect Life Sciences, New York, NY USA

**Keywords:** Artificial intelligence (AI), Translation, Linguistic validation (LV), Clinical outcome assessments (COAs), Patient-reported outcomes (PROs), Intellectual property (IP), Cultural adaptation, Intellectual property (IP)

## Abstract

**Background:**

While there has been much discussion around the use of Artificial Intelligence (AI) for multilingual translations in other areas, recommendations pertaining specifically to the use of AI in the context of Clinical Outcome Assessment (COA) translation, linguistic validation, and electronic migration within clinical trials are lacking. Without published recommendations or guidelines, stakeholders involved in the COA translation process may be hesitant to explore or include AI. To address this gap, the AI Working Group of the ISOQOL TCA-SIG conducted a study to assess the landscape of AI in this specific context aimed at proposing recommendations for potential implementation of AI in COA translation, linguistic validation and electronic migration processes.

**Methodology:**

The study consisted of three parts: (1) a literature review targeting studies using AI in COA translation; (2) a survey among relevant stakeholders assessing perceptions of AI use in COA translation; and (3) interviews with AI subject matter experts (SMEs).

**Results:**

Survey responses were received from a total of 50 individuals from a wide variety of stakeholder groups, including COA copyright holders, representatives from pharmaceutical company COA/HEOR teams, respondents holding roles associated with the COA translation, eCOA, and AI industries, and authors of the 2005 ISPOR task force article on linguistic validation methodology. Survey data provided detailed feedback regarding the appropriateness of using AI during all reviewed process steps. Results of the literature review and AI expert interviews provided additional depth and nuance, allowing for the generation of detailed recommendations covering the use of AI within linguistic validation and eCOA migration processes.

**Conclusions:**

When assessing the potential use of AI tools within the linguistic validation process, it is important to consider not only the capabilities of the technology, but also the degree to which use of AI may or may not align with the spirit and intent of existing linguistic validation guidelines. The recommendations included in this manuscript are designed to balance considerations of technological capability and improved efficiency with concerns related to intellectual property protection, data privacy/security, and the goal of keeping patients at the center of outcomes research.

**Supplementary Information:**

The online version contains supplementary material available at 10.1186/s41687-026-01012-5.

## Background

Artificial intelligence (AI) is a rapidly evolving area transforming both the translation industry and the healthcare industry as a whole. AI, as defined through discussion and examination of existing definitions by the AI Working Group of the ISOQOL Translation and Cultural Adaptation Special Interest Group (TCA-SIG), is a technology that enables computers and machines to simulate human intelligence and problem-solving capabilities. The U.S. Food and Drug Administration (FDA) defines AI as “a machine-based system that can, for a given set of human-defined objectives, make predictions, recommendations, or decisions influencing real or virtual environments. AI systems use machine- and human-based inputs to perceive real and virtual environments; abstract such perceptions into models through analysis in an automated manner; and use model inference to formulate options for information or action” [1].

**Fig. 1:**
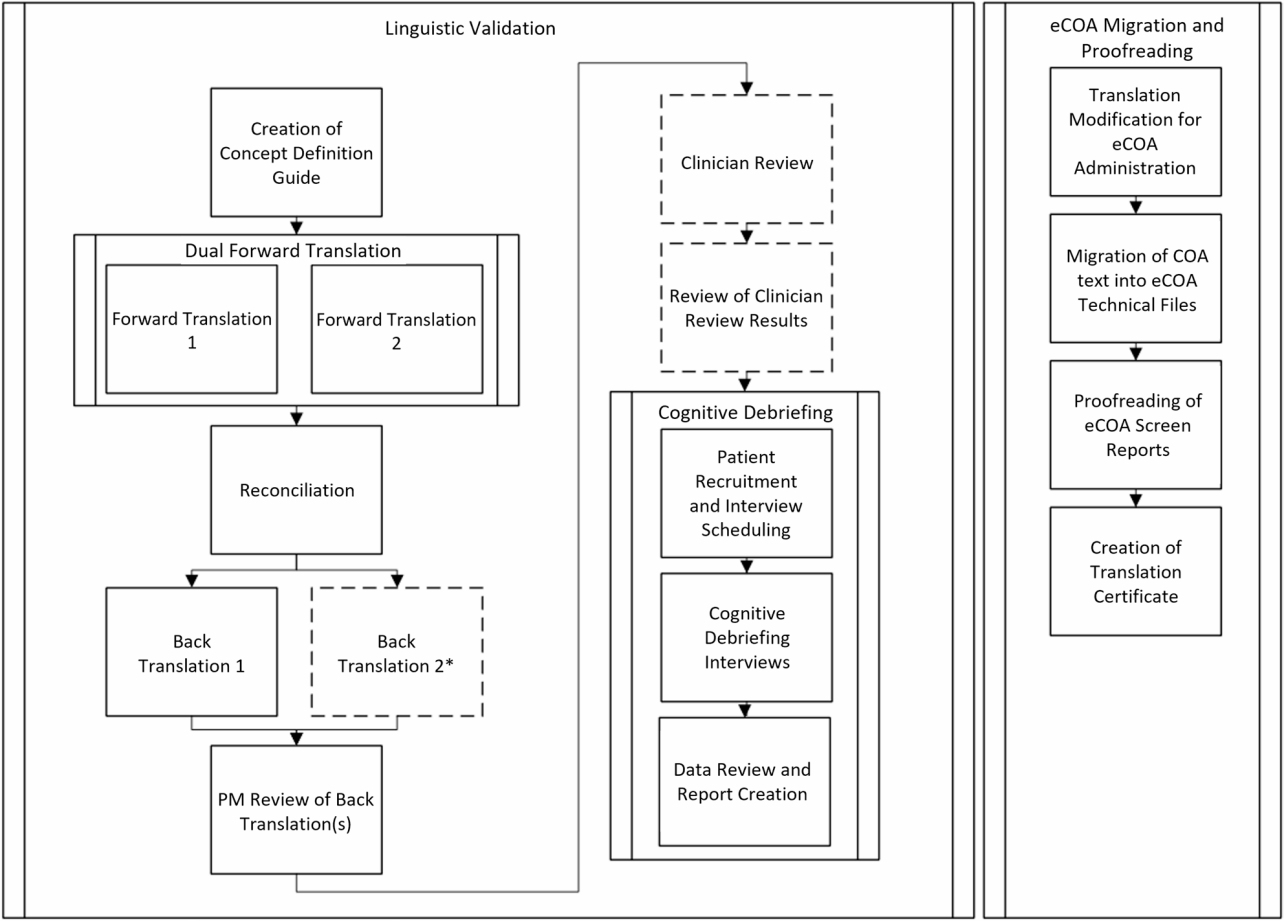
Linguistic Validation Process Map. Dotted lines indicate processes that may be optional for some projects. *LV best practice guidelines recommend at least one back-translation. Some LV providers or methodologies may include two back-translations in this step

In its Patient-Focused Drug Development guidance, FDA notes that “poorly translated survey instruments can prevent researchers from collecting data comparable to that of surveys in the source (original) language” [2]. To maintain conceptual equivalence and cultural appropriateness across languages and countries when translating clinical outcome assessments (COAs), a complex linguistic validation (LV) methodology was codified by Wild et al. in 2005. This methodology relies on input from multiple translators, reviewers, and proofreaders, and requires interviewing native-speaking patients with a relevant health status or condition to ensure translations are adequately comprehended by the target population [3]. Figure 1 provides a detailed map of key LV process steps which has been expanded to include related processes such as eCOA migration, while Appendix A provides brief descriptions of these steps. Approaches consistent with this have since been accepted as best practice by regulatory bodies, outcomes research professionals, and clinical trialists.

As translation and LV of COAs is a time- and cost-intensive endeavor, potential exists to leverage AI to make the process more efficient [4]. However, this potential may be difficult to realize without thorough assessment of AI’s capabilities and risks in relation to translation quality and cultural appropriateness. Another key concern is protecting the intellectual property of COA copyright holders; the European Medicines Agency (EMA) cautions that Large Language Model (LLM) outputs could potentially “reveal sensitive information included in datasets used for training” and “infringe on other legal rights, such as copyright” [5].

While much discussion about using AI for multilingual translations in other areas exists, recommendations pertaining specifically to AI use in the context of COA translation, LV, and electronic migration within clinical trials are lacking. Presently, the most widely referenced guidelines and recommendations associated with COA translation processes do not cover the potential use of AI [3, 6–8]. Without published recommendations or guidelines specific to each of the many steps in the LV process, stakeholders involved in the COA translation process may be hesitant to explore or include AI; conversely, a lack of guidance may encourage potentially inappropriate AI use, jeopardizing the validity of those newly translated measures. To address this gap, the ISOQOL TCA-SIG AI Working Group conducted a study to assess the landscape of AI in this specific context aimed at proposing well-defined and granular recommendations for potential AI implementation in COA translation, LV and electronic migration processes.

## Methods

The study consisted of three parts: (1) a literature review targeting studies using AI in COA translation; (2) a survey among relevant stakeholders assessing perceptions of AI use in COA translation; and (3) interviews with AI subject matter experts (SMEs).

### Literature review

A search was conducted in PubMed from inception to July 5, 2024, with a combination of search terms for COAs, AI, and translation. No language or other restrictions were applied. The full search strategy is reported in Supplement 1 [9]. Studies were included if they used AI for translating at least one COA, or if they referenced AI use in the translation of COAs.

### Survey

A core survey containing 27 items was drafted and finalized via group discussion Supplement 2. Questions focused on capabilities of AI in supporting specific LV process steps, whether introduction of AI would be a positive or negative development, security of IP, how respondents perceived the views of other key stakeholder groups, and potential effects of AI on project costs and timelines. Nine items elicited free text responses related to the above topics. Free text response prompts appeared after each of the 18 structured items.

The AI Working Group noted that regardless of AI’s technical capability to contribute to COA/eCOA translation tasks, adoption of the technology was currently limited by fears that key stakeholder groups would not find AI use acceptable. To explore this concept further, respondents were asked whether they believed COA copyright holders, pharmaceutical sponsors, and regulatory bodies would find AI involvement in specific process steps acceptable or unacceptable. For COA copyright holders and pharmaceutical sponsors, the AI Working Group could then compare actual views of these stakeholders against their views as perceived by respondents in different stakeholder categories.

Following completion of the core survey, three additional versions were created, each targeting a specific stakeholder group Supplement 2. These groups included (1) COA copyright holders, (2) individuals working within pharmaceutical companies’ COA or HEOR departments, and (3) members of the original authorship group of the 2005 ISPOR task force article by Wild et al., widely seen as the foundational publication for current standard practices [3].

Surveys were sent to 100 recipients, including 24 copyright holders, 9 pharmaceutical employees focused on COA/HEOR, 2 regulators, 5 members of the original ISPOR task force not involved as co-authors of this manuscript, 12 employees of language service providers (LSPs), 20 freelance translators experienced in COA translation, 8 AI specialists, 2 IRB representatives, 4 employees of HEOR consulting firms, 12 employees of eCOA providers, and 2 leaders of the ISOQOL Scientific Program Committee for the 2025 annual conference. Any recipients who did not receive one of the three variations described above were sent the core survey. The surveys were completed online during October and November 2024.

### Interviews with AI SMEs

After initial survey results review, the AI Working Group agreed that while the responses were relevant, useful, and representative of a wide variety of COA experts, there was insufficient technical input on which to base recommendations. Accordingly, five in-depth semi-structured interviews with AI SMEs were conducted. These interviews were completed in January and February of 2025, and participants included AI experts from IQVIA, Lionbridge, RWS, and TransPerfect, companies which provide translation, LV, and technology services, and WOLK.ai, a software and AI services company. Each participant was asked the same series of questions focused on AI security, intellectual property, and AI capabilities within the LV processes.

## Results

### Literature review

A total of 553 abstracts were identified, assigned and screened. No screened manuscripts were relevant according to pre-defined criteria. Other literature germane to COAs, AI and translation like conference presentations and white papers were identified via working group recommendations (*n* = 6) and are summarized here.

### COA translation comparison/evaluation studies

Several studies investigated how AI-based translation compares to human translation in the context of COA translation. Using Metric for Evaluation of Translation with Explicit ORdering (METEOR) scoring, Lu and colleagues compared Arabic, Vietnamese, Italian, Hungarian, Malay, and Dutch translations produced by LLMs (GPT-4, GPT-3.5 and Google Translate) to human translations of portions of the Breast-Q and Face-Q PRO measures. Their findings suggested that while LLMs can provide high-quality translations to support human translators, substituting human translators with machine translation (MT) entirely is not advisable [10]. Similarly, Kunst and colleagues compared AI-supported (Google Translate and GPT-3.5) and human translations of a personality inventory. They proposed a framework describing four potential levels of integration for AI in cross-cultural research, varying by language type, ranging from AI as a preliminary forward translation to full AI translation with human review [11]. Wolk and colleagues developed a semi-automatic semantic evaluation metric to assess the quality of Polish translations of 150 items from the Patient-Reported Outcome Measurement Information System (PROMIS). Their results indicated that the human-aided translation evaluation metric (HMEANT) aligned most closely with expert human judgments, suggesting potential utility for AI-based models in COA translation quality control [12].

### AI in specific COA translation process steps

Other research focused on AI’s potential involvement in specific COA translation steps. Casale and colleagues leveraged Chat GPT-4o to compare source text to back-translated text categorizing it as identical, equivalent, or needing review while also generating contextual comments for non-identical items. Across a sample of approximately 1,000 words, AI outperformed human translators with over 5 years of experience, though the authors emphasized the need for further testing before large scale adoption [13]. Olesa and colleagues explored generative AI applications in the context of concept elaboration – the process of creating clear and comprehensive explanation of COA concepts. Using Chat GPT-4o, they conducted a blinded, five-category comparison of AI and human generated elaborations across various COA types and sizes. Although human outputs generally demonstrated higher quality, AI achieved comparable performance in some categories. The authors concluded that a human-in-the-loop approach currently represents the most reliable strategy to ensure accuracy and completeness in COA concept elaboration [14].

### Perceptions and implementation challenges related to AI use in the context of COA translation

Poepsel and colleagues investigated professional attitudes toward AI integration within COA translation and LV processes. Their survey of LV professionals (*N* = 52) and linguists (*N* = 83) representing 60 languages found that although both groups did see benefit in using machine translation as part of the COA translation process, fair levels of resistance and hesitancy existed. The authors emphasized the need for regulatory guidance and the establishment of industry-wide best practices to support responsible use of AI in COA translation [15].

### Summary of literature

Taken together, these studies illustrate the emerging and multifaceted role of AI in COA translation and LV. While AI systems show promise in improving efficiency, consistency and quality assessment, the evidence consistently supports maintaining a human-in-the-loop model. Continued methodological research coupled with clear regulatory and ethical frameworks is essential to ensure responsible adoption of AI in this evolving field.

### Survey results

Survey responses were received from 50 individuals (response rate 50%). 15 (30%) were COA copyright holders, 5 (10%) were representatives from pharmaceutical company COA/HEOR teams, 28 (56%) were from respondents holding roles associated with the COA translation, eCOA, and AI industries, and 2 (4%) were 2005 ISPOR task force article authors. No responses were received from regulatory body representatives. One survey respondent later joined the AI Working Group but did not participate as an author. No other members completed the survey. Respondents could skip questions, and so response rates for individual questions ranged from 84% (42/50) to 100% (50/50).

Respondents could report the organization with which they were affiliated. 29 respondents did so; organizations included the Art of Diversity Translation, Critical Path Institute, Eli Lilly and Company, EORTC, EuroQol, FACITtrans, GSK, ICON, IQVIA, Lionbridge, MD Anderson Cancer Center, Medable, Medical College of Wisconsin, Novartis, Oxford University Innovation, RWS, Suvoda, University of Notre Dame, University of North Carolina at Charlotte, VeLo Language Services, and YPrime. No organization had more than 2 respondents.

#### Capabilities of AI

For the following analysis, all respondents were grouped together to generate an industry-wide snapshot of AI use perceptions. A more granular analysis of stakeholder group perceptions follows in subsequent sections. Respondents were asked to assess whether AI was capable of effectively completing key elements of the LV process. Results indicated strong agreement that AI was partially capable of translating COAs into languages commonly required for clinical trials in terms of translation accuracy (41/50, 82%) and cultural appropriateness (33/48, 69%). For languages used more rarely in trials, respondent agreement was moderate; a 47% (21/45) plurality believed AI to be partially capable for both language categories. eCOA migration and proofreading tasks were viewed as particularly suitable for AI, with 84% (42/50) of respondents indicating that AI would be partially or fully capable of improving these processes. Table 1 presents the full respondent group results.

**Table 1:** Capabilities of AI for translation and eCOA migration/proofreading

Capability of AI	Survey Responses
Accurately translating COAs into languages commonly required for clinical trials (e.g., French, Spanish, German, Portuguese, Chinese, Japanese)	Not at all capable: 8% Partially capable: 82% Fully capable: 6% Not sure: 4%
Accurately translating COAs into all languages that may be required for use in clinical trials, including those used more rarely (e.g., Zulu, Malayalam, Tagalog)	Not at all capable: 24.5% Partially capable: 47% Fully capable: 4% Not sure: 24.5%
Creating culturally appropriate translations of COAs into languages commonly required for clinical trials	Not at all capable: 14.5% Partially capable: 69% Fully capable: 2% Not sure: 14.5%
Creating culturally appropriate translations of COAs into all languages that may be required for use in clinical trials, including those used more rarely	Not at all capable: 33% Partially capable: 47% Fully capable: 0% Not sure: 20%
Improving the eCOA migration and proofreading process	Not at all capable: 2% Partially capable: 54% Fully capable: 30% Not sure: 14%

In assessing AI’s capability of effectively managing the CD process (Table 2), respondents reported that AI was either partially or fully capable of performing administrative tasks like patient recruitment and interview scheduling (33/47, 70%), data review, and report preparation (41/49, 84%). However, results indicate much lower confidence in AI actually performing CD interviews with patients, with only 44% (22/50) of respondents endorsing “partially” or “fully” capable.

Free-text responses indicated a nuanced view of AI’s LV capabilities, noting that while AI can be “a useful and helpful tool” in the process, “the accuracy, cultural sensitivity, and patient-centered nature required in COA translation and validation still heavily rely on human expertise and empathy.”

**Table 2:** Capabilities of AI for the cognitive debriefing process

Capability of AI	Survey Responses
Improving the process of patient recruitment and interview scheduling for cognitive debriefing interviews	Not at all capable: 8% Partially capable: 43% Fully capable: 28% Not sure: 21%
Improving the process of data review and report preparation for cognitive debriefing interviews	Not at all capable: 6% Partially capable: 53% Fully capable: 31% Not sure: 10%
Effectively conducting cognitive debriefing interviews with patients	Not at all capable: 42% Partially capable: 42% Fully capable: 2% Not sure: 14%

Respondents were asked how AI use would impact project costs and timelines (Table 3). Results overwhelmingly indicated that AI use would reduce or significantly reduce timelines (86%, 38/44). There was less consensus regarding how AI use would affect project costs; 62% (26/42) thought costs would either decrease or significantly decrease, while 24% (10/42) believed they would increase or significantly increase. Free-text comments showed a key driver of this difference was the idea that “it is expensive to develop, test and maintain AI systems,” which may offset cost reductions elsewhere.

**Table 3:** AI impact on costs and timelines

Impact of AI	Survey Responses
On project timelines	Significantly Lengthen: 2% Lengthen: 5% No Impact: 7% Reduce: 72.5% Significantly Reduce: 13.5%
On project costs	Significantly Increase: 2% Increase: 22% No Impact: 14% Decrease: 57% Significantly Decrease: 5%

#### Positive and negative evaluations of AI use

In addition to providing their assessment of AI’s capabilities, respondents evaluated whether specific COA/eCOA process steps would be positively affected, negatively affected, or not affected by AI use. Table 4 shows the full respondent group results.

**Table 4:** Positive and negative perceptions of AI use in linguistic validation process steps

Process Step	Survey Responses
Creation of Concept Definition / Concept Elaboration document	Positively affected: 68% Negatively affected: 22% Not affected: 10%
Dual forward translations	Positively affected: 62% Negatively affected: 33.5% Not affected: 4.5%
Reconciliation of forward translations	Positively affected: 45.5% Negatively affected: 34% Not affected: 20.5%
Back-translation	Positively affected: 63% Negatively affected: 28% Not affected: 9%
Project Manager review and evaluation of back-translation	Positively affected: 34% Negatively affected: 43% Not affected: 23%
Proofreading	Positively affected: 67.5% Negatively affected: 17.5% Not affected: 15%
Review of clinician review results	Positively affected: 43% Negatively affected: 35% Not affected: 22%
Cognitive debriefing interviews	Positively affected: 22% Negatively affected: 63% Not affected: 15%
Review of cognitive debriefing data	Positively affected: 60% Negatively affected: 23% Not affected: 17%
Modification of COA translations for eCOA administration	Positively affected 64% Negatively affected 16% Not affected 20%
eCOA migration	Positively affected 83% Negatively affected 4% Not affected 13%
eCOA proofreading	Positively affected 71% Negatively affected 18% Not affected 11%
Creation of translation certificate	Positively affected 78% Negatively affected 4% Not affected 18%

Of the 13 process steps reviewed, 11 were considered positively affected by AI by a majority or plurality of respondents. Only 2 process steps were judged to be negatively affected by AI: *Project Manager review and evaluation of back-translations* (20/47, 43% negative), and *cognitive debriefing interviews* (29/46, 63% negative).

While most process steps were judged to be positively aligned with AI usage, free-text comments indicated that these complex processes would require significant human intervention and guidance. A representative comment indicated that “[…] AI has a place in the translation, linguistic validation and eCOA migration process […] I do not think it can totally replace humans in these steps, but it can be a valuable aide which should improve quality, efficiency, and hopefully timelines.” Several respondents suggested that pilot projects be designed to allow for direct comparisons of translation accuracy and cultural appropriateness.

In addition to reviewing process steps individually, respondents assessed whether introducing AI into the COA/eCOA translation, LV, and eCOA migration processes would be a negative or positive development (Table 5). Most respondents viewed AI as a positive development, with 73% (36/49) characterizing their views as either “somewhat positive” or “extremely positive.”

**Table 5:** Overall assessment of AI use in COA/eCOA translation, LV, and eCOA migration

Rating	Core Survey	Pharma Sponsors	Copyright Holders	ISPOR Thought Leaders	Overall
Extremely Negative	4 (14%)	0	0	0	**4 (8%)**
Somewhat Negative	1 (4%)	0	0	1 (50%)	**2 (4%)**
Neither negative nor positive	5 (18%)	0	2 (14%)	0	**7 (14%)**
Somewhat positive	14 (50%)	2 (40%)	10 (71%)	1 (50%)	**27 (55%)**
Extremely positive	4 (14%)	3 (60%)	2 (14%)	0	**9 (18%)**
	**(28)**	**(5)**	**(14)**	**(2)**	**(49)**

#### Assessment of stakeholder group views (perceived and actual)

Respondents were next asked whether they believed key stakeholder groups (copyright holders, pharmaceutical sponsors, and regulators) would find AI use acceptable for a reduced set of 6 process steps. The reduced set was selected to reduce respondent burden. Use of different survey iterations as described above permitted direct comparison of perceived and actual responses for the copyright holder and pharmaceutical sponsors groups, preventing the target groups from self-rating on their own perceived opinions.

#### Copyright holders

Table 6 presents the results for perceived and actual copyright holder responses to AI use within various steps of the COA/eCOA translation process. For 5 of 6 process steps, copyright holders’ “acceptable” percentage was higher than the full group’s perceived “acceptable” percentage. While copyright holder responses showed greater openness to acceptability of AI use than the full respondent set, only 3 of 6 steps were judged “acceptable” for AI use by a majority of copyright holders.

**Table 6:** COA copyright holders, perceived vs. actual views

Process Step	Perceived Copyright Holder Responses	Actual Copyright Holder Responses	Differential
Forward translation process	Acceptable: 26.5% Unacceptable: 26.5% Not sure: 47%	Acceptable: 31% Unacceptable: 23% Not sure: 46%	Acceptable: +4.5% Unacceptable: -3.5% Not sure: -1%
Back-translation process	Acceptable: 35% Unacceptable: 18% Not sure: 47%	Acceptable: 31% Unacceptable: 23% Not sure: 46%	Acceptable: -4% Unacceptable: +5% Not sure: -1%
Patient recruitment and interview scheduling for cognitive debriefing interviews	Acceptable: 35% Unacceptable: 32.5% Not sure: 32.5%	Acceptable: 62% Unacceptable: 0% Not sure: 38%	Acceptable: +27% Unacceptable: -32.5% Not sure: +5.5%
Conducting cognitive debriefing interviews with patients	Acceptable 3% Unacceptable 65% Not sure 32%	Acceptable 8% Unacceptable 54% Not sure 38%	Acceptable: + 5% Unacceptable: - 11% Not sure: + 6%
Data review and report preparation for cognitive debriefing interviews	Acceptable 38% Unacceptable 24% Not sure 38%	Acceptable 62% Unacceptable 15% Not sure 23%	Acceptable: + 24% Unacceptable: - 9% Not sure: - 15%
eCOA migration and proofreading	Acceptable 47% Unacceptable 15% Not sure 38%	Acceptable 70% Unacceptable 15% Not sure 15%	Acceptable: + 23% Unacceptable: -0% Not sure: - 23%

#### Pharmaceutical sponsors

Table 7 presents the results for perceived and actual pharmaceutical sponsor responses. Actual responses from pharmaceutical representatives were quite open to AI use, and pharmaceutical sponsors’ “acceptable” percentage was higher than the full group’s perceived “acceptable” percentage for all 6 process steps. Overall, AI involvement was deemed acceptable by 80% to 100% (4/5 and 5/5) of pharmaceutical respondents for 5 of 6 steps. As with all respondent groups, AI conducting CD interviews was the exception, judged as acceptable by only 40% (2/5) of pharmaceutical representatives.

In free-text responses, non-pharmaceutical respondents noted that “sponsors are generally open to any advancement […] as long as it is proven, in a data-driven way, to be reliable and valid.” However, it was also noted that sponsors “would expect robust human validation to ensure quality, as accuracy and cultural appropriateness are critical in patient-centered assessments.”

Pharmaceutical representatives themselves indicated openness to AI use in free-text comments, particularly “selective targeted use of AI [which] should result in overall improvements,” such as using AI for one of the two required forward translations. They cautioned, however, that there would be “serious concerns about handing over the entire process, or even chunks of the process to AI without human oversight,” and that they “would want to make sure that from a regulatory process [perspective] we won’t have data rejected due to AI being used.”

**Table 7:** Pharmaceutical sponsors (COA/HEOR), perceived vs. actual views

Process Step	Perceived Pharmaceutical Sponsor Responses	Actual Pharmaceutical Sponsor Responses	Differential
Forward translation process	Acceptable: 51% Unacceptable: 17% Not sure: 32%	Acceptable: 80% Unacceptable: 0% Not sure: 20%	Acceptable: +29% Unacceptable: -17% Not sure: -12%
Back-translation process	Acceptable: 62.5% Unacceptable: 12.5% Not sure: 25%	Acceptable: 80% Unacceptable: 0% Not sure: 20%	Acceptable: +17.5% Unacceptable: -12.5% Not sure: -5%
Patient recruitment and interview scheduling for cognitive debriefing interviews	Acceptable: 55% Unacceptable: 18% Not sure: 27%	Acceptable: 100% Unacceptable: 0% Not sure: 0%	Acceptable: +45% Unacceptable: -18% Not sure: -27%
Conducting cognitive debriefing interviews with patients	Acceptable: 32% Unacceptable: 32% Not sure: 36%	Acceptable: 40% Unacceptable: 60% Not sure: 0%	Acceptable: +8% Unacceptable: +28% Not sure: -36%
Data review and report preparation for cognitive debriefing interviews	Acceptable: 51% Unacceptable: 15% Not sure: 34%	Acceptable: 80% Unacceptable: 20% Not sure: 0%	Acceptable: +29% Unacceptable: +5% Not sure: -34%
eCOA migration and proofreading	Acceptable: 58% Unacceptable 10% Not sure: 32%	Acceptable: 80% Unacceptable: 20% Not sure: 0%	Acceptable: +22% Unacceptable: +10% Not sure: -32%

#### Regulatory representatives

While survey responses from regulatory representatives were not provided, all respondents were asked whether they thought regulatory bodies would find COA/eCOA deliverables involving AI use acceptable or unacceptable. The results (Table 8) indicate pervasive uncertainty regarding regulatory thinking on the topic, with a plurality of respondents “not sure” what regulators would consider acceptable for 5 of 6 process steps. The exception again was AI conducting CD interviews, which 57% (26/46) of respondents felt would be unacceptable to regulatory bodies.

In free-text comments, views were notably mixed. Some respondents noted that “within regulatory bodies such as the FDA and EMA, AI use is [considered to be] inevitable,” and that “regulatory bodies will be cautiously open to the use of AI,” while others suggested that regulatory bodies will likely be “over-careful,” and would require “ample evidence to be comfortable with AI, especially where the intersection of AI and patient care comes into play.”

**Table 8:** Regulatory bodies (e.g., FDA, EMA) perceived views

Process Step	Perceived Regulatory Responses
Forward translation process	Acceptable: 25% Unacceptable: 30% Not sure: 45%
Back-translation process	Acceptable: 30% Unacceptable: 25% Not sure: 45%
Patient recruitment and interview scheduling for cognitive debriefing interviews	Acceptable: 32% Unacceptable: 28% Not sure: 40%
Conducting cognitive debriefing interviews with patients	Acceptable: 4% Unacceptable: 57% Not sure: 39%
Data review and report preparation for cognitive debriefing interviews	Acceptable: 30% Unacceptable: 28% Not sure: 42%
eCOA migration and proofreading	Acceptable: 41% Unacceptable: 15% Not sure: 44%

#### Copyright, security, and intellectual property

To assess concerns related to IP rights and inappropriate or illegal COA use, respondents were asked about their level of concern with AI usage from an IP perspective. When asked how concerned they would be about the use of AI translation processes having a negative impact on the protection of COA IP, 57% (27/47) selected “concerned” or “extremely concerned,” while 36% (17/47) selected “somewhat concerned” and 6% (3/47) selected “not at all concerned.” When asked how concerned they would be about the use of AI translation processes contributing to inappropriate or illegal use of COAs from a copyright perspective, 62% (28/45) selected “concerned” or “extremely concerned,” while 27% (12/45) selected “somewhat concerned” and 11% (5/45) selected “not at all concerned.” Copyright holders showed somewhat more concern about IP protection than the total respondent group, with a majority selecting “extremely concerned” (54%, 7/13) in response to the second question.

### Subject matter expert interview results

#### Security and copyright issues

AI SMEs were advised of the concerns identified in the survey results regarding AI negatively impacting protection of COA IP and were asked to address EMA’s 2024 guidance on the topic [5]. All interviewees agreed that where appropriate procedures are followed, such as using dedicated or proprietary AI deployments that prevent data from leaving the AI system and implementing data storage and sharing restrictions, AI use presented a low risk of contributing to copyright infringement. Several interviewees noted that the standard LV process may introduce more risk than AI due to the involvement of 10+ human collaborators per language.

#### Linguistic validation AI process capabilities

Interview participants were asked to assess the capability of AI to accurately translate COAs into languages rarely used in clinical trials and to assess AI capabilities to create culturally appropriate, rather than technically accurate, translations for such languages. Interviewees widely agreed that rarely used languages are more challenging for AI to translate effectively due to the lack of large corpora for training the systems. This training requires significant investment, but interviewees agreed that the practice is advancing and compared it to the current use of professional human translators in less frequently used languages, where identifying qualified linguists can sometimes be difficult.

In assessing AI capabilities associated with CD interviews, interview participants were more hesitant. While all agreed that AI’s capability to perform interviews exists and may eventually match trained human interviewers, participants noted that in interview settings “the human aspect plays a role” and that interviewer empathy and interpersonal dynamics are important to ensure successful interviews. Participants provided many potential AI use cases beyond conducting interviews, including transcript creation, data population and evaluation, identification of patterns within and across interviews, and suggesting probing questions.

## Discussion

When assessing the potential use of AI tools within the LV process, it is important to consider not only the technology’s capabilities, but the degree to which AI use may or may not align with the spirit and intent of existing LV guidelines. The 2005 *Principles of Good Practice* noted the need for a robust methodology “given the risks that poor translation methods can present to research data.” The authors further described their goals of “ensuring that the translation is comprehensible to the general or patient population,” “ensuring conceptual equivalence between the source and target language versions and between all translations,” and avoiding creation of “a translation that does not respect the normal speech patterns and colloquialisms of the target culture” [3].

These survey results clearly demonstrate respondents’ concerns about poor alignment between AI and the intent of the current guidelines, and about the prospect of eliminating or significantly reducing human involvement in a process that has patients at its center. The results indicate that AI is perceived by the respondent group to be potentially useful within many elements of the COA LV process, but not fully capable of completing tasks adequately without human involvement. Results also indicate that key stakeholder groups like copyright holders and pharmaceutical sponsors are consistently more likely to consider AI use acceptable than the total respondent set believed them to be.

After reviewing the survey and interview results, as well as literature and presentations detailing successful case studies, it is the opinion of the AI Working Group that AI use to enhance and facilitate LV process steps does not conflict with the aims or outcomes of existing guidelines if managed responsibly. With humans remaining in the loop at each step, AI can be used to make existing processes more accurate, powerful, and efficient, particularly for languages commonly used in clinical trials. All the same, the appropriateness of AI use for any particular step should be carefully considered and contextualized within overall project methodology and constrained by the complexity of that step. That is, AI use may be more appropriate in multi-step methodologies already featuring multiple points of human quality review, and more appropriately deployed for lower complexity (e.g., eCOA migration) vs. higher complexity (e.g., cognitive debriefing) steps. The recommendations developed through this work are, in part, intended as a necessary and facilitating precursor to appropriate targeting of LV process steps for testing with AI. They provide data-based constraints for subsequent experimental work by other researchers, helping ensure that future approaches to integrating AI are both aligned with the established objectives of LV and likely to succeed.

In some ways, the iterative process utilized by AI systems is similar to the complex, iterative process of LV. AI use involves multiple layered and precisely targeted interactions intended to achieve specific outputs (e.g., one prompted interaction creating the initial translation, another assessing cultural nuances, another comparing translations to one another). This approach aligns well with the spirit and function of the existing LV process, which iteratively guides the translation through multiple inputs and quality assessments to ensure the collection of high-quality data.

**Fig. 2:**
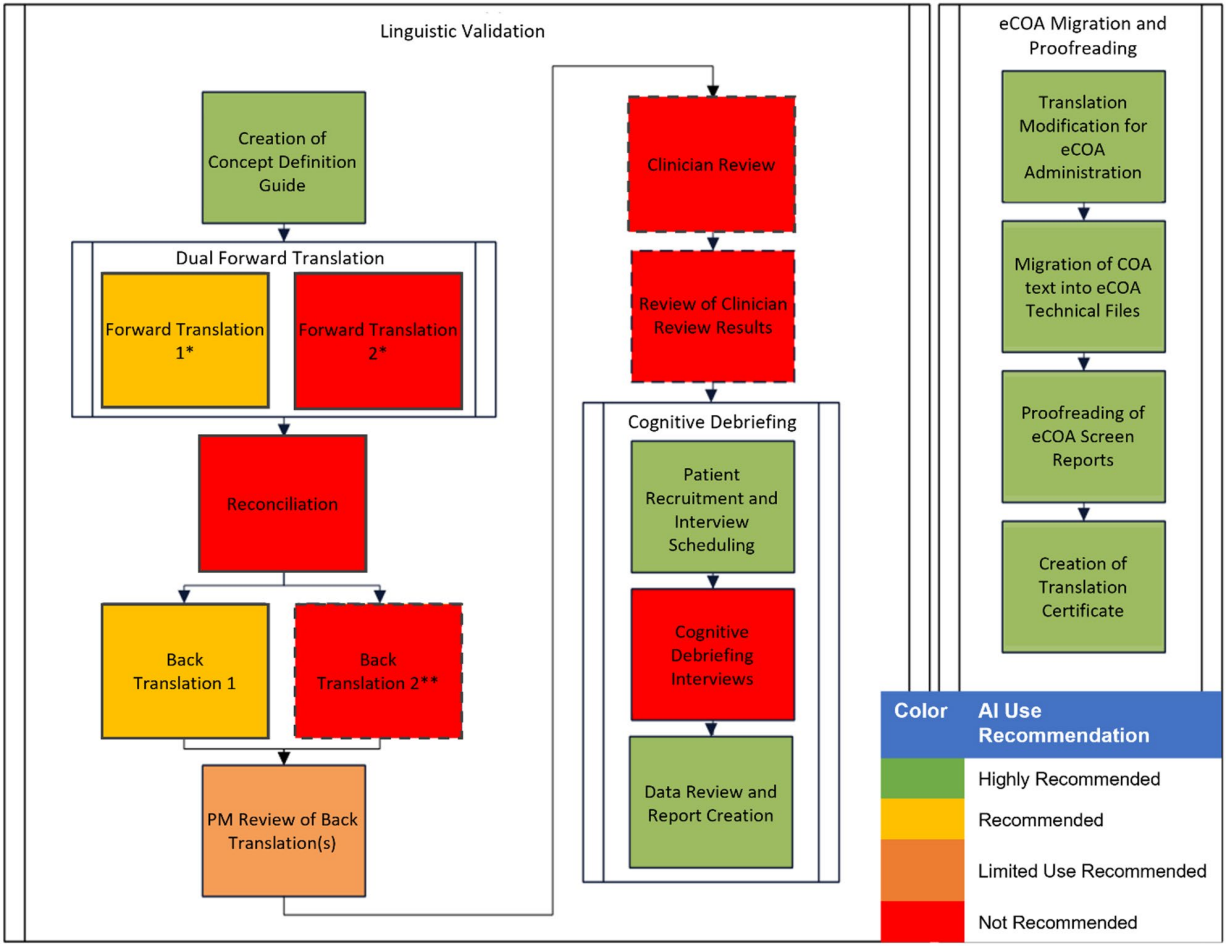
Modified LV process Map with AI Use Recommendations Represented by Color. Dotted lines indicate processes that may be optional for some projects. *AI use is only recommended for one of the two translations in the Dual Forward Translation step. **LV best practice guidelines recommend at least one back-translation. Some LV providers or methodologies may include two back-translations in this step

Table 9 contains recommendations for potential AI involvement in the LV process derived from the results of literature review, survey responses, expert interviews, and AI Working Group discussion. Synthesizing these data sources, the working group found them to be largely aligned, with the literature review demonstrating effective AI pilot projects while the survey and interviews provided generally positive capability assessments. In cases where data sources were in conflict regarding particular process recommendations (e.g., “Review and evaluation of back-translation against the source COA” had positive results in the literature, but a negative survey result), the AI Working Group assessed the results holistically to produce suitable recommendations. Figure 2 presents an updated linguistic validation process map, modified by the inclusion of colors corresponding to our recommendations for each process step.

**Table 9:** Recommendations for potential AI involvement in the linguistic validation process. Process steps are presented in chronological order

Process Step	Recommendation for AI Involvement	Comments	Origin of Recommendation
Creation of Concept Definition Guide	Use is highly recommended, under human oversight	While human review and confirmation is required, AI can be of use in generating draft item concept definitions and storing relevant elements for re-use.	Literature Review, Survey Results (68% positive), AI Working Group Discussion
Dual forward translation	Use is recommended, under human oversight	One human-generated translation and one AI/MT translation, rather than two human-generated translations.	Literature Review, Survey Results (62% positive), Expert Interviews, AI Working Group Discussion
Reconciliation of dual forward translations	Use is not recommended	This process likely needs to remain with humans in order to achieve the goals of current guidelines.	Survey results (45.5% positive), AI Working Group Discussion
Back-translation (single or dual)	Use is recommended, under human oversight	One human-generated back-translation and one AI/MT back-translation may bring added insight and accuracy to the process.	Literature Review, Survey Results (63% positive), AI Working Group Discussion
Review and evaluation of back-translation against the source COA	Limited use is recommended, under human oversight	Despite low survey scores, results of the literature review indicate that AI can contribute to the comparative review between source and back-translations at a high level [11].	Literature Review, Survey Results (34% positive), AI Working Group Discussion
Clinician review	Use is not recommended	This process likely needs to remain with humans in order to achieve the goals of current guidelines.	AI Working Group Discussion
Review of clinician review results	Use is not recommended	This process likely needs to remain with humans in order to achieve the goals of current guidelines.	Survey Results (43.5% positive), AI Working Group Discussion
Patient recruitment and interview scheduling for cognitive debriefing interviews	Highly recommended, under human oversight	This is an administrative task well suited to AI completion. Patient privacy issues should be taken into careful consideration, as well as ensuring that the respondent sample is suitably diverse from a sociodemographic perspective.	Survey Results (60% positive), Expert Interviews, AI Working Group Discussion
Conducting cognitive debriefing interviews with patients	Use is not recommended	While AI may be useful in generating interview guides, probes, etc., the interview process likely needs to remain with humans in order to achieve the goals of current guidelines. Significant concerns about effectiveness and patient perception were raised, which may improve in the future but are not recommended at present.	Survey Results (22% positive), Expert Interviewers, AI Working Group Discussion
Data review and report preparation for cognitive debriefing interviews	Highly recommended, under human oversight	AI can be very useful in preparing and analyzing data; human review of this output is still required. Patient privacy issues should be taken into careful consideration.	Survey Results (60% positive), Expert Interviewers, AI Working Group Discussion
Modification of COA translations for electronic (eCOA) administration	Highly recommended, under human oversight	AI can analyze source COA and eCOA files to identify modifications and assist in making relevant eCOA updates to existing COA translations.	Survey Results (64% positive), AI Working Group Discussion
Migration of translated COA text into eCOA technical files	Highly recommended, under human oversight	This highly technical task is an excellent match for AI capabilities.	Survey Results (83% positive), AI Working Group Discussion
Proofreading of eCOA screen reports	Highly recommended, under human oversight	Proofreading can likely be largely automated using AI. Human oversight should remain.	Survey Results (71% positive), AI Working Group Discussion
Creation of translation certificate	Highly recommended, under human oversight	This is an administrative task well suited to AI completion. Process steps listed in translation certificates should note if AI was used to perform the listed step.	Survey Results (78% positive), AI Working Group Discussion

### Security and copyright recommendations

COAs frequently contain copyrighted IP. Survey results, particularly copyright holder responses, identified that AI usage could trigger concerns about maintenance of IP rights and inappropriate or illegal use of these measures. Table 10 presents best practice recommendations for protection of COA data while using AI.

**Table 10:** SME recommendations and rationales for ensuring secure AI systems

SME Recommendation	Rationale
Use only dedicated or proprietary deployments of AI	Use of dedicated cloud-hosted or proprietary on-premises AI deployments ensures that data does not flow out of the AI system. Open generative AI chatbots (e.g., ChatGPT) do not fit this description. Commercially available LLMs from reputable providers (e.g., Microsoft, Amazon, etc.) are acceptable from a security standpoint if a copy of the LLM is hosted within a secure environment.
Controlled storage or no storage of data	If input data are either securely controlled in an effectively encrypted memory or purged from the system after the AI interaction has ended, the risk of data leakage is significantly reduced.
No sharing of data with third parties	Data flow should be fully controlled while data are in use, and data should not leave the dedicated AI deployment.

### Limitations

While this paper gathered data and made recommendations based on literature review, custom surveys distributed to industry COA experts, and in-depth interviews with AI experts, the AI Working Group did not directly test the use of AI on LV process steps. We encourage industry stakeholders to build on our recommendations through responsible tests of AI implementation and continue growing the literature on this important topic. Although beyond the scope of this study, subsequent research could adopt an evaluation framework that integrates translation metrics like the ones cited in the literature review with human judgment.

## Conclusion

While it is clear that, as noted by FDA, “AI technologies have the potential to transform healthcare by deriving new and important insights from the vast amount of data generated during the delivery of healthcare every day” [16], there is lack of consensus around AI use in the context of COA translations specifically. The AI Working Group of the ISOQOL TCA-SIG sought to make recommendations to fill this gap via literature review, survey research, expert interviews and group discussion. Much like the original LV guidelines, these recommendations do not prescribe a set of rigid procedures, but rather set out principles of good practice. AI is rapidly evolving, and capabilities which may not currently be reliable could very well become so in the coming years. As a result, these recommendations are meant to serve as a foundation and set of guidelines encouraging responsible AI use within the linguistic validation process, while ensuring that human interaction and review remain present for all steps.

## Appendix

Appendix A: Descriptions of linguistic validation process steps.

**Table:** 

Creation of Concept Definition Guide	A document providing critical conceptual information about the COA and its wording, and possible translation issues, for use by all stakeholders and participants in the LV process. Can convey notes from the instrument developer or copyright holder, as well.
Dual Forward Translation	Two linguists independently generate translations of a COA in the target language.
Reconciliation	The translations from the previous step are reviewed and merged into a single translation, with linguists selecting the most appropriate components of each independent forward translation.
Back-translation	A blinded conversion of the translation back into the source language by an independent linguist. LV best practice guidelines recommend at least one back-translation. Some LV providers or methodologies may include two back-translations in this step.
PM Review of Back-translation(s)	Comparison of the back-translation to the source text to ensure that source concepts are accurately translated and conveyed. If translation or conceptual issues are identified, the translation is updated accordingly.
Clinician Review	A clinician in an appropriate area of patient care for the COA reviews the translation for conceptual or translation problems. This step occurs only in some LV methodologies or for certain types of COAs.
Review of Clinician Review Results	The clinician review is analyzed by a project manager (PM) or other expert reviewer, and translation updates are made as needed based on the findings. This step occurs only when Clinician review has been performed.
Patient Recruitment and Interview Scheduling	Identification of a suitable sample of patients for cognitive debriefing, contacting of patients and scheduling of in-person or remote interviews.
Cognitive Debriefing Interviews	A trained interviewer conducts cognitive debriefing interviews with the recruited sample of patients to assess comprehension and cultural relevance of the COA translations.
Data Review and Report Creation	Cognitive debriefing feedback from the patient sample and interviewer is reviewed by a PM or other expert reviewer. If translation or conceptual issues are identified, the translation is updated accordingly. Re-testing of updated translations with the same or another patient sample is performed as needed.
Translation Modification for eCOA Administration	The wording of a translation is updated to be as appropriate as possible for electronic administration.
Migration of eCOA Text into eCOA Technical Files	The draft eCOA text is moved into eCOA technical files, which prepares it for correct implementation and display in an electronic format.
Proofreading of eCOA Screen Reports	Screen reports with the translation displayed as it would be in electronic format are generated and reviewed for accuracy and adherence to eCOA best-practices by appropriate expert reviewers.
Creation of Translation Certificate	A translation certificate detailing process steps and deliverables is created.

## Supplementary Information

Below is the link to the electronic supplementary material.

Supplementary Material 1

Supplementary Material 2

## Data Availability

The datasets used and/or analyzed during the current study are available from the corresponding author on reasonable request.

## References

[1] U.S. Food and Drug Administration. (n.d.). Artificial intelligence and medical products. US Food and Drug Administration. https://www.fda.gov/science-research/science-and-research-special-topics/artificial-intelligence-and-medical-products. Accessed 23 Jan 2025

[2] U.S. Food and Drug Administration. Patient-focused drug development: methods to identify what is important to patients. Guidance for industry, Food and Drug Administration staff, and other stakeholders. Silver Spring (MD): Food and Drug Administration (US), Department of Health and Human Services; 2022. Available from. https://www.fda.gov/regulatory-information/search-fda-guidance-documents/patient-focused-drug-development-methods-identify-what-important-patients. Accessed 23 Jan 2025

[3] Wild D, Grove A, Martin M, Eremenco S, McElroy S, Verjee-Lorenz A, Erickson P (2005) Principals of good practice for the translation and cultural adaptation process for patient-reported outcomes (PRO) measures: report of the ISPOR task force for translation and cultural adaptation. Value Health 8:94–104. 10.1111/j.1524-4733.2005.04054.x 15804318 10.1111/j.1524-4733.2005.04054.x

[4] Williams H (2024) Harmonising linguistic validation with AI: Precision, efficiency, and the human touch in patient-reported outcome translation. Med Writ 33(1):66–69. 10.56012/lxmw9690

[5] European Medicines Agency (2024) Guiding principles on the use of large language models in regulatory science and for medicines regulatory activities. https://www.ema.europa.eu. Accessed 23 Jan 2025.

[6] Wild D, Eremenco S, Mear I, Martin M, Houchin C, Gawlicki M, Hareendran A, Wiklund I, Chong LY, von Maltzahn R, Cohen L, Molsen E (2009) Multinational trials—Recommendations on the translations required, approaches to using the same Language in different countries, and the approaches to support pooling the data: the ISPOR Patient-Reported outcomes translation and linguistic validation good research practices task force report. Value Health 12(4):430–440. 10.1111/j.1524-4733.2008.00471.x 19138309 10.1111/j.1524-4733.2008.00471.x

[7] Eremenco S, Pease S, Mann S, Berry P (2018) Patient-Reported outcome (PRO) consortium translation process: consensus development of updated best practices. J Patient-Reported Outcomes 2(1):12. 10.1186/s41687-018-0037-6 10.1186/s41687-018-0037-6 PMC5934912 29757299

[8] McKown S, Acquadro C, Anfray C, Arnold B, Eremenco S, Giroudet C, Martin M, Weiss D (2020) Good practices for the translation, cultural adaptation, and linguistic validation of clinician-reported outcome, observer-reported outcome, and performance outcome measures. J Patient Rep Outcomes 2020(41):89. 10.1186/s41687-020-00248-z 10.1186/s41687-020-00248-z PMC7642163 33146755

[9] Terwee CB, Jansma EP, Riphagen II, de Vet HC (2009) Development of a methodological pubmed search filter for finding studies on measurement properties of measurement instruments. Qual Life Res 18(8). 10.1007/s11136-009-9528-5 10.1007/s11136-009-9528-5 PMC2744791 19711195

[10] Lu SC, Xu C, Kaur M, Edelen MO, Pusic A, Gibbons C (2025) Can machine translation match human expertise? Quantifying the performance of large language models in the translation of patient-reported outcome measures (PROMs)*.* J Patient Rep Outcomes. 2025;9(1):94. 10.1186/s41687-025-00926-w 10.1186/s41687-025-00926-w PMC12297096 40711496

[11] Kunst JR, Bierwiaczonek K (2023) Utilizing AI questionnaire translations in cross-cultural and intercultural research: insights and recommendations. Int J Intercult Relat 97:101888. 10.1016/j.ijintrel.2023.101888

[12] Wolk K, Glinkowski W, Żukowska A (2018) Enhancing the assessment of (Polish) translation in PROMIS using statistical, semantic, and neural network metrics. In: WorldCIST’18. Advances in Intelligent Systems and Computing, vol. 746. Cham: Springer International Publishing; 2018. p. 351–66.366). 10.1007/978-3-319-77712-2_34

[13] Casale S, Johnson M, Nolte K (2024) Gen AI powered precision: revolutionizing comparative review in clinical outcome assessment localization. Value Health 27(12):S50. 10.1016/j.jval.2024.10.266

[14] Olesa ES, Johnson M, Agüero F, Bodzer A, Casale S, Costa S, Jimenez KE, Nolte K, O’Gara L (2024) Transforming concept elaboration in COA localization: A gen AI based approach. Value Health 27(12):S42. 10.1016/j.jval.2024.10.225

[15] Poepsel T, Israel R, Nolde A, Hadjidemetriou C, Browning R, Ramsey P, Delgaram-Nejad O, McCullough E, McKown S (2024) Perceptions of the feasibility of AI-Based machine translation for linguistic validation. Value Health 27(6):S270–271. 10.1016/j.jval.2024.03.1494

[16] D U.S. Food and Drug Administration, Health Canada, & Medicines and Healthcare products Regulatory Agency (2023). Predetermined change control plans for machine learning-enabled medical devices: guiding principles. 2023.https://www.fda.gov/medical-devices/software-medical-device-samd/good-machine-learning-practice-medical-device-development-guiding-principles. Accessed 23 Jan 2025

