# Supplementary material for: Use of AI within COA linguistic validation and eCOA migration processes: analysis and good practice recommendations
Source: J Patient Rep Outcomes. 2026 Feb 6;10:34. doi: 10.1186/s41687-026-01012-5 (PMC12936314; doi:10.1186/s41687-026-01012-5)
Supplement: Supplementary file 1 — Supplementary Material 1 [file 41687_2026_1012_MOESM1_ESM.docx]

*Supplement 1: PubMed Literature Review Search Strategy [16]*

| #1 | PROMs (search terms extracted from the COSMIN search filter for finding studies on measurement properties)  HR-PRO[tiab] OR HRPRO[tiab] OR HRQL[tiab] OR HRQoL[tiab] OR QL[tiab] OR QoL[tiab] OR quality of life[tw] OR life quality[tw] OR health index*[tiab] OR health indices[tiab] OR health profile*[tiab] OR health status[tw] OR ((patient[tiab] OR self[tiab] OR child[tiab] OR parent[tiab] OR carer[tiab] OR proxy[tiab]) AND ((report[tiab] OR reported[tiab] OR reporting[tiab]) OR (rated[tiab] OR rating[tiab] OR ratings[tiab]) OR based[tiab] OR (assessed[tiab] OR assessment[tiab] OR assessments[tiab]))) OR ((disability[tiab] OR function[tiab] OR functional[tiab] OR functions[tiab] OR subjective[tiab] OR utility[tiab] OR utilities[tiab] OR wellbeing[tiab] OR well being[tiab]) AND (index[tiab] OR indices[tiab] OR instrument[tiab] OR instruments[tiab] OR measure[tiab] OR measures[tiab] OR questionnaire[tiab] OR questionnaires[tiab] OR profile[tiab] OR profiles[tiab] OR scale[tiab] OR scales[tiab] OR score[tiab] OR scores[tiab] OR status[tiab] OR survey[tiab] OR surveys[tiab])) |
| --- | --- |
| #2 | Additional search terms for other COAs  "patient reported outcome"[tiab] OR "patient reported outcomes"[tiab] OR "patient-reported outcome"[tiab] OR "patient-reported outcomes"[tiab] OR "clinician reported outcome"[tiab] OR "clinician reported outcomes"[tiab] OR "clinician-reported outcome"[tiab] OR "clinician-reported outcomes"[tiab] OR "observer reported outcome"[tiab] OR "observer reported outcomes"[tiab] OR "observer-reported outcome"[tiab] OR "observer-reported outcomes"[tiab] OR "performance outcome"[tiab] OR "performance outcomes"[tiab] OR "performance based outcome"[tiab] OR "performance based outcomes"[tiab] OR "performance-based outcome"[tiab] OR "performance-based outcomes"[tiab] |
| #3 | Search terms for AI  "Artificial intelligence"[tiab] OR AI[tiab] OR "Machine intelligence"[tiab] OR "Computational intelligence"[tiab] OR "Cognitive automation"[tiab] OR "Machine learning"[tiab] OR "Deep learning"[tiab] |
| #4 | Translation[tiab] |

Final search: (#1 OR #2) AND #3 AND #4.
Search date: July 5, 2024
Number of abstracts found: 553
