# Supplementary material for: Use of AI within COA linguistic validation and eCOA migration processes: analysis and good practice recommendations
Source: J Patient Rep Outcomes. 2026 Feb 6;10:34. doi: 10.1186/s41687-026-01012-5 (PMC12936314; doi:10.1186/s41687-026-01012-5)
Supplement: Supplementary file 2 — Supplementary Material 2 [file 41687_2026_1012_MOESM2_ESM.pdf]

Appendix B **: CORE Survey**

Introduction

While there has been much discussion around the use of AI for translations in other areas, recommendations pertaining specifically to the use of AI in the context of COA translation and linguistic validation are lacking. The purpose of this survey is to address the lack of consensus around the use of AI in the context of COA translations and linguistic validation and explore the appropriateness and viability of AI use within various stages of COA and eCOA translation, linguistic validation, and electronic (eCOA) migration processes.

For the purposes of this survey, we are using the following definition of AI:

"Artificial intelligence, or AI, is technology that enables computers and machines to simulate human intelligence and problem-solving capabilities."

A more detailed definition of AI, which reinforces the one above, is provided by FDA:

"Artificial Intelligence (AI) is a machine-based system that can, for a given set of human-defined objectives, make predictions, recommendations, or decisions influencing real or virtual environments. AI systems use machine- and human-based inputs to perceive real and virtual environments; abstract such perceptions into models through analysis in an automated manner; and use model inference to formulate options for information or action. AI includes machine learning, which is a set of techniques that can be used to train AI algorithms to improve performance of a task based on data."

Thank you for participating in this survey. Your feedback is appreciated and important. The free text boxes are optional; please complete them only if you wish to. The survey should take about 10-15 minutes, and your feedback is confidential.

AI Capabilities and Appropriateness of Use

1. Do you believe that AI is capable of accurately translating COAs into **languages commonly required** for clinical trials (e.g., French, Spanish, German, Portuguese, Chinese, Japanese, etc.)?

- ◯ Not at all capable
- ◯ Partially capable
- ◯ Fully capable
- ◯ Not sure

If you have additional comments about this question, feel free to enter them here:

2. Do you believe that AI is capable of accurately translating COAs into **all languages that may be required** for use in clinical trials, including those used more rarely (e.g., Zulu, Malayalam, Tagalog, etc.)?

- ◯ Not at all capable
- ◯ Partially capable
- ◯ Fully capable
- ◯ Not sure

If you have additional comments about this question, feel free to enter them here:

3. Do you believe that AI is capable of creating **culturally appropriate** translations of COAs into **languages commonly required** for clinical trials (e.g., French, Spanish, German, Portuguese, Chinese, Japanese, etc.)?

- ◯ Not at all capable
- ◯ Partially capable
- ◯ Fully capable
- ◯ Not sure

If you have additional comments about this question, feel free to enter them here:

4. Do you believe that AI is capable of creating **culturally appropriate** translations of COAs into **all languages that may be required** for use in clinical trials, including those used more rarely (e.g., Zulu, Malayalam, Tagalog, etc.)?

- ◯ Not at all capable
- ◯ Partially capable
- ◯ Fully capable
- ◯ Not sure

If you have additional comments about this question, feel free to enter them here:

5. Do you believe that AI is capable of improving the process of patient recruitment and interview scheduling for cognitive debriefing interviews?

- ◯ Not at all capable
- ◯ Partially capable
- ◯ Fully capable
- ◯ Not sure

If you have additional comments about this question, feel free to enter them here:

6. Do you believe that AI is capable of effectively conducting cognitive debriefing interviews with patients?

- ◯ Not at all capable
- ◯ Partially capable
- ◯ Fully capable
- ◯ Not sure

If you have additional comments about this question, feel free to enter them here:

7. Do you believe that AI is capable of improving the process of data review and report preparation for cognitive debriefing interviews?

- ◯ Not at all capable
- ◯ Partially capable
- ◯ Fully capable
- ◯ Not sure

If you have additional comments about this question, feel free to enter them here:

8. Do you believe that AI is capable of improving the eCOA migration and proofreading process?

- ◯ Not at all capable
- ◯ Partially capable
- ◯ Fully capable
- ◯ Not sure

If you have additional comments about this question, feel free to enter them here:

9. Overall, do you believe that the introduction of AI into the COA/eCOA translation, linguistic validation, and eCOA migration processes would be a negative or positive development?

- ◯ Extremely negative
- ◯ Somewhat negative
- ◯ Neither negative nor positive
- ◯ Somewhat positive
- ◯ Extremely positive

If you have additional comments about this question, feel free to enter them here:

10. Do you believe that AI systems involved in COA/eCOA translation, linguistic validation, and eCOA migration processes should be closed systems (i.e., restricted to a company- specific network)?

- ◯ Yes
- ◯ No
- ◯ Not sure

If you have additional comments about this question, feel free to enter them here:

11. Please evaluate whether the COA / eCOA process steps below would be positively or negatively affected by the use of AI, or unaffected by it. If you are not sure how a given process step would be affected, you may skip that row in the table below.

	Positively affected by use of AI	Negatively affected by use of AI	Not affected by use of AI

Creation of Concept Definition/Concept Elaboration document			
Dual forward translation			
Reconciliation of forward translations			
Back-translation			
Project Manager review and evaluation of back-translation			
Proofreading			
Review of clinician review results			
Cognitive debriefing interviews with patients			
Review of cognitive debriefing interview results			
Modification of COA translations for electronic (eCOA) administration			
Migration of translated COA text into eCOA technical files			
Proofreading of eCOA screen reports			
Creation of translation certificate			

Other (please identify any missing process steps and evaluate)

12. Please provide any additional comments you have regarding the use of AI within the COA/eCOA translation, linguistic validation, and eCOA migration processes.

13. Is any COA/eCOA process step that could be affected by the use of AI missing from the questions above? Please add any comments about missing steps here.

Intellectual Property and Data Confidentiality; Copyright Holder Reaction

14. COA measures frequently contain copyrighted intellectual property. How concerned would you be about the use of AI translation processes having a negative impact on the protection of intellectual property of COA measures?

- ◯ Not at all concerned
- ◯ Somewhat concerned
- ◯ Concerned
- ◯ Extremely concerned

If you have additional comments about this question, feel free to enter them here:

15. How concerned would you be about the use of AI translation processes contributing to inappropriate or illegal use of COA measures from a copyright perspective?

- ◯ Not at all concerned
- ◯ Somewhat concerned
- ◯ Concerned
- ◯ Extremely concerned

If you have additional comments about this question, feel free to enter them here:

16. For the following processes, please rate whether you think **COA copyright holders or developers** would find COA / eCOA deliverables involving the use of AI acceptable or unacceptable:

	Acceptable for use	Unacceptable for use	Not sure
Forward translation process	◯	◯	◯
Back-translation process	◯	◯	◯
Patient recruitment and interview scheduling for cognitive debriefing interviews	◯	◯	◯
Conducting cognitive debriefing interviews with patients	◯	◯	◯
Data review and report preparation for cognitive debriefing interviews	◯	◯	◯
eCOA migration and proofreading	◯	◯	◯

17. For the same processes, if you have additional comments about the acceptability of COA / eCOA deliverables involving the use of AI from the perspective of **COA copyright holders or developers**, please enter them here:

Forward translation process	
Back-translation process	
Patient recruitment and interview scheduling for cognitive debriefing interviews	
Conducting cognitive debriefing interviews with patients	
Data review and report preparation for cognitive debriefing interviews	
eCOA migration and proofreading	

18. Please provide your comments regarding how you believe **COA copyright holders or developers** would react to the use of AI in COA translation, back-translation, cognitive debriefing, or eCOA migration processes.

Regulatory Reaction

19. For the following processes, please rate whether you think **regulatory bodies** (e.g., FDA, EMA) would find COA / eCOA deliverables involving the use of AI acceptable or unacceptable:

	Acceptable for use	Unacceptable for use	Not sure
Forward translation process	◯	◯	◯
Back-translation process	◯	◯	◯
Patient recruitment and interview scheduling for cognitive debriefing interviews	◯	◯	◯
Conducting cognitive debriefing interviews with patients	◯	◯	◯
Data review and report preparation for cognitive debriefing interviews	◯	◯	◯
eCOA migration and	◯	◯	◯

proofreading

20. For the same processes, if you have additional comments about the acceptability of COA / eCOA deliverables involving the use of AI from the perspective of **regulatory bodies** (e.g., FDA, EMA), please enter them here:

Forward translation process	
Back-translation process	
Patient recruitment and interview scheduling for cognitive debriefing interviews	
Conducting cognitive debriefing interviews with patients	
Data review and report preparation for cognitive debriefing interviews	
eCOA migration and proofreading	

21. Please provide your comments regarding how you believe **regulatory bodies** would react to the use of AI in COA translation, back-translation, cognitive debriefing, or eCOA migration processes.

Pharmaceutical Sponsor Reaction

22. For the following processes, please rate whether you think **pharmaceutical sponsors** (COA scientists, HEOR professionals, etc.) would find COA / eCOA deliverables involving the use of AI acceptable or unacceptable:

	Acceptable for use	Unacceptable for use	Not sure
Forward translation process	○	○	○
Back-translation process	○	○	○
Patient recruitment and interview scheduling for cognitive debriefing interviews	○	○	○
Conducting cognitive debriefing interviews with patients	○	○	○
Data review and report preparation for cognitive debriefing interviews	○	○	○
eCOA migration and proofreading			

23. For the same processes, if you have additional comments about the acceptability of COA / eCOA deliverables involving the use of AI from the perspective of **pharmaceutical sponsors** (COA scientists, HEOR professionals, etc.), please enter them here:

Forward translation process	
Back-translation process	
Patient recruitment and interview scheduling for cognitive debriefing interviews	
Conducting cognitive debriefing interviews with patients	
Data review and report preparation for cognitive debriefing interviews	
eCOA migration and proofreading	

24. Please provide your comments regarding how you believe **pharmaceutical sponsors** (COA scientists, HEOR professionals, etc.) would react to the use of AI in COA translation, back-translation, cognitive debriefing, or eCOA migration processes.

Costs and Timelines

25. How do you think that use of AI during the COA/eCOA translation, linguistic validation, and eCOA migration processes would impact project timelines?

- Significantly Lengthen
- Lengthen
- No impact
- Reduce
- Significantly Reduce

If you have additional comments about this question, feel free to enter them here:

26. How do you think that use of AI during the COA/eCOA translation, linguistic validation, and eCOA migration processes would impact project costs?

- Significantly Increase
- Increase
- No impact
- Decrease
- Significantly Decrease

If you have additional comments about this question, feel free to enter them here:

27. Please provide your comments on how specifically costs and timelines may be affected by use of AI in COA translation, back-translation, cognitive debriefing, or eCOA migration processes.

28. Thanks for your participation! Please provide the name of the company or organization you represent below.

Thank you very much for completing this survey!

Appendix C : **COA Copyright Holders Survey**

Introduction

While there has been much discussion around the use of AI for translations in other areas, recommendations pertaining specifically to the use of AI in the context of COA translation and linguistic validation are lacking. The purpose of this survey is to address the lack of consensus around the use of AI in the context of COA translations and linguistic validation and explore the appropriateness and viability of AI use within various stages of COA and eCOA translation, linguistic validation, and electronic (eCOA) migration processes.

For the purposes of this survey, we are using the following definition of AI:

"Artificial intelligence, or AI, is technology that enables computers and machines to simulate human intelligence and problem-solving capabilities."

A more detailed definition of AI, which reinforces the one above, is provided by FDA:

"Artificial Intelligence (AI) is a machine-based system that can, for a given set of human-defined objectives, make predictions, recommendations, or decisions influencing real or virtual environments. AI systems use machine- and human-based inputs to perceive real and virtual environments; abstract such perceptions into models through analysis in an automated manner; and use model inference to formulate options for information or action. AI includes machine learning, which is a set of techniques that can be used to train AI algorithms to improve performance of a task based on data."

Thank you for participating in this survey. Your feedback is appreciated and important. The free text boxes are optional; please complete them only if you wish to. The survey should take about 10-15 minutes, and your feedback is confidential.

AI Capabilities and Appropriateness of Use

1. Do you believe that AI is capable of accurately translating COAs into **languages commonly required** for clinical trials (e.g., French, Spanish, German, Portuguese, Chinese, Japanese, etc.)?

- ◯ Not at all capable
- ◯ Partially capable
- ◯ Fully capable
- ◯ Not sure

If you have additional comments about this question, feel free to enter them here:

2. Do you believe that AI is capable of accurately translating COAs into **all languages that may be required** for use in clinical trials, including those used more rarely (e.g., Zulu, Malayalam, Tagalog, etc.)?

- ◯ Not at all capable
- ◯ Partially capable
- ◯ Fully capable
- ◯ Not sure

If you have additional comments about this question, feel free to enter them here:

3. Do you believe that AI is capable of creating **culturally appropriate** translations of COAs into **languages commonly required** for clinical trials (e.g., French, Spanish, German, Portuguese, Chinese, Japanese, etc.)?

- ◯ Not at all capable
- ◯ Partially capable
- ◯ Fully capable
- ◯ Not sure

If you have additional comments about this question, feel free to enter them here:

4. Do you believe that AI is capable of creating **culturally appropriate** translations of COAs into **all languages that may be required** for use in clinical trials, including those used more rarely (e.g., Zulu, Malayalam, Tagalog, etc.)?

- ◯ Not at all capable
- ◯ Partially capable
- ◯ Fully capable
- ◯ Not sure

If you have additional comments about this question, feel free to enter them here:

5. Do you believe that AI is capable of improving the process of patient recruitment and interview scheduling for cognitive debriefing interviews?

- ◯ Not at all capable
- ◯ Partially capable
- ◯ Fully capable
- ◯ Not sure

If you have additional comments about this question, feel free to enter them here:

6. Do you believe that AI is capable of effectively conducting cognitive debriefing interviews with patients?

- ◯ Not at all capable
- ◯ Partially capable
- ◯ Fully capable
- ◯ Not sure

If you have additional comments about this question, feel free to enter them here:

7. Do you believe that AI is capable of improving the process of data review and report preparation for cognitive debriefing interviews?

- ◯ Not at all capable
- ◯ Partially capable
- ◯ Fully capable
- ◯ Not sure

If you have additional comments about this question, feel free to enter them here:

8. Do you believe that AI is capable of improving the eCOA migration and proofreading process?

- ◯ Not at all capable
- ◯ Partially capable
- ◯ Fully capable
- ◯ Not sure

If you have additional comments about this question, feel free to enter them here:

9. Overall, do you believe that the introduction of AI into the COA/eCOA translation, linguistic validation, and eCOA migration processes would be a negative or positive development?

- ◯ Extremely negative
- ◯ Somewhat negative
- ◯ Neither negative nor positive
- ◯ Somewhat positive
- ◯ Extremely positive

If you have additional comments about this question, feel free to enter them here:

10. Do you believe that AI systems involved in COA/eCOA translation, linguistic validation, and eCOA migration processes should be closed systems (i.e., restricted to a company- specific network)?

- ◯ Yes
- ◯ No
- ◯ Not sure

If you have additional comments about this question, feel free to enter them here:

11. Please evaluate whether the COA / eCOA process steps below would be positively or negatively affected by the use of AI, or unaffected by it. If you are not sure how a given process step would be affected, you may skip that row in the table below.

	Positively affected by use of AI	Negatively affected by use of AI	Not affected by use of AI

Creation of Concept Definition/Concept Elaboration document	◯	◯	◯
Dual forward translation	◯	◯	◯
Reconciliation of forward translations	◯	◯	◯
Back-translation	◯	◯	◯
Project Manager review and evaluation of back-translation	◯	◯	◯
Proofreading	◯	◯	◯
Review of clinician review results	◯	◯	◯
Cognitive debriefing interviews with patients	◯	◯	◯
Review of cognitive debriefing interview results	◯	◯	◯
Modification of COA translations for electronic (eCOA) administration	◯	◯	◯
Migration of translated COA text into eCOA technical files	◯	◯	◯
Proofreading of eCOA screen reports	◯	◯	◯
Creation of translation certificate	◯	◯	◯

Other (please identify any missing process steps and evaluate)

12. Please provide any additional comments you have regarding the use of AI within the COA/eCOA translation, linguistic validation, and eCOA migration processes. Is any COA/eCOA process step that could be affected by the use of AI missing from the questions above? Please add any comments about missing steps here.

13. Is any COA/eCOA process step that could be affected by the use of AI missing from the questions above? Please add any comments about missing steps here.

Intellectual Property and Data Confidentiality; Copyright Holder Reaction

14. COA measures frequently contain copyrighted intellectual property. How concerned would you be about the use of AI translation processes having a negative impact on the protection of intellectual property of COA measures?

- Not at all concerned
- Somewhat concerned
- Concerned
- Extremely concerned

If you have additional comments about this question, feel free to enter them here:

15. How concerned would you be about the use of AI translation processes contributing to inappropriate or illegal use of COA measures from a copyright perspective?

- Not at all concerned
- Somewhat concerned
- Concerned
- Extremely concerned

If you have additional comments about this question, feel free to enter them here:

16. As a **COA copyright holder or developer**, please rate whether you would find COA / eCOA deliverables involving the use of AI acceptable or unacceptable for the following processes:

	Acceptable for use	Unacceptable for use	Not sure
Forward translation process	○	○	○
Back-translation process	○	○	○
Patient recruitment and interview scheduling for cognitive debriefing interviews	○	○	○
Conducting cognitive debriefing interviews with patients	○	○	○
Data review and report preparation for cognitive debriefing interviews	○	○	○
eCOA migration and proofreading	○	○	○

17. For the same processes, if you have additional comments about the acceptability of COA / eCOA deliverables involving the use of AI, please enter them here:

Forward translation process

Back-translation process

Patient recruitment and interview scheduling for cognitive debriefing interviews

Conducting cognitive debriefing interviews with patients	
Data review and report preparation for cognitive debriefing interviews	
eCOA migration and proofreading	

18. Please provide your comments regarding how you as a **COA copyright holder or developer** would react to the use of AI in COA translation, back-translation, cognitive debriefing, or eCOA migration processes.

Regulatory Reaction

19. For the following processes, please rate whether you think **regulatory bodies** (e.g., FDA, EMA) would find COA / eCOA deliverables involving the use of AI acceptable or unacceptable:

	Acceptable for use	Unacceptable for use	Not sure
Forward translation process	○	○	○
Back-translation process	○	○	○
Patient recruitment and interview scheduling for cognitive debriefing interviews	○	○	○
Conducting cognitive debriefing interviews with patients	○	○	○
Data review and report preparation for cognitive debriefing interviews	○	○	○
eCOA migration and proofreading	○	○	○

20. For the same processes, if you have additional comments about the acceptability of COA / eCOA deliverables involving the use of AI from the perspective of **regulatory bodies** (e.g., FDA, EMA), please enter them here:

Forward translation process	
Back-translation process	
Patient recruitment and interview scheduling for cognitive debriefing interviews	

Conducting cognitive debriefing interviews with patients	
Data review and report preparation for cognitive debriefing interviews	
eCOA migration and proofreading	

21. Please provide your comments regarding how you believe **regulatory bodies** would react to the use of AI in COA translation, back-translation, cognitive debriefing, or eCOA migration processes.

Pharmaceutical Sponsor Reaction

22. For the following processes, please rate whether you think **pharmaceutical sponsors** (COA scientists, HEOR professionals, etc.) would find COA / eCOA deliverables involving the use of AI acceptable or unacceptable:

	Acceptable for use	Unacceptable for use	Not sure
Forward translation process	○	○	○
Back-translation process	○	○	○
Patient recruitment and interview scheduling for cognitive debriefing interviews	○	○	○
Conducting cognitive debriefing interviews with patients	○	○	○
Data review and report preparation for cognitive debriefing interviews	○	○	○
eCOA migration and proofreading	○	○	○

23. For the same processes, if you have additional comments about the acceptability of COA / eCOA deliverables involving the use of AI from the perspective of **pharmaceutical sponsors** (COA scientists, HEOR professionals, etc.), please enter them here:

Forward translation process	
Back-translation process	
Patient recruitment and interview scheduling for cognitive debriefing interviews	

Conducting cognitive debriefing interviews with patients	
Data review and report preparation for cognitive debriefing interviews	
eCOA migration and proofreading	

24. Please provide your comments regarding how you believe **pharmaceutical sponsors** would react to the use of AI in COA translation, back-translation, cognitive debriefing, or eCOA migration processes.

Costs and Timelines

25. How do you think that use of AI during the COA/eCOA translation, linguistic validation, and eCOA migration processes would impact project timelines?

- ◯ Significantly Lengthen
- ◯ Lengthen
- ◯ No impact
- ◯ Reduce
- ◯ Significantly Reduce

If you have additional comments about this question, feel free to enter them here:

26. How do you think that use of AI during the COA/eCOA translation, linguistic validation, and eCOA migration processes would impact project costs?

- ◯ Significantly Increase
- ◯ Increase
- ◯ No impact
- ◯ Decrease
- ◯ Significantly Decrease

If you have additional comments about this question, feel free to enter them here:

27. Please provide your comments on how specifically costs and timelines may be affected by use of AI in COA translation, back-translation, cognitive debriefing, or eCOA migration processes.

28. Thanks for your participation! Please provide the name of the company or organization you represent below.

Thank you very much for completing this survey!

Appendix D : **Pharmaceutical Sponsors Survey**

Introduction

While there has been much discussion around the use of AI for translations in other areas, recommendations pertaining specifically to the use of AI in the context of COA translation and linguistic validation is lacking. The purpose of this survey is to address the lack of consensus around the use of AI in the context of COA translations and linguistic validation, and explore the appropriateness and viability of AI use within various stages of COA and eCOA translation, linguistic validation, and electronic (eCOA) migration processes.

For the purposes of this survey, we are using the following definition of AI:

"*Artificial intelligence, or AI, is technology that enables computers and machines to simulate human intelligence and problem-solving capabilities*."

A more detailed definition of AI, which reinforces the one above, is provided by FDA:

"Artificial Intelligence (AI) is a machine-based system that can, for a given set of human-defined objectives, make predictions, recommendations, or decisions influencing real or virtual environments. AI systems use machine- and human-based inputs to perceive real and virtual environments; abstract such perceptions into models through analysis in an automated manner; and use model inference to formulate options for information or action. AI includes machine learning, which is a set of techniques that can be used to train AI algorithms to improve performance of a task based on data."

Thank you for participating in this survey. Your feedback is appreciated and important. The free text boxes are optional; please complete them only if you wish to. The survey should take about 10-15 minutes, and your feedback is confidential.

AI Capabilities and Appropriateness of Use

1. Do you believe that AI is capable of accurately translating COAs into **languages commonly required** for clinical trials (e.g., French, Spanish, German, Portuguese, Chinese, Japanese, etc.)?

- ◯ Not at all capable
- ◯ Partially capable
- ◯ Fully capable
- ◯ Not sure

If you have additional comments about this question, feel free to enter them here:

2. Do you believe that AI is capable of accurately translating COAs into **all languages that may be required** for use in clinical trials, including those used more rarely (e.g., Zulu, Malayalam, Tagalog, etc.)?

- ◯ Not at all capable
- ◯ Partially capable
- ◯ Fully capable
- ◯ Not sure

If you have additional comments about this question, feel free to enter them here:

3. Do you believe that AI is capable of creating **culturally appropriate** translations of COAs into **languages commonly required** for clinical trials (e.g., French, Spanish, German, Portuguese, Chinese, Japanese, etc.)?

- ◯ Not at all capable
- ◯ Partially capable
- ◯ Fully capable
- ◯ Not sure

If you have additional comments about this question, feel free to enter them here:

4. Do you believe that AI is capable of creating **culturally appropriate** translations of COAs into **all languages that may be required** for use in clinical trials, including those used more rarely (e.g., Zulu, Malayalam, Tagalog, etc.)?

- ◯ Not at all capable
- ◯ Partially capable
- ◯ Fully capable
- ◯ Not sure

If you have additional comments about this question, feel free to enter them here:

5. Do you believe that AI is capable of improving the process of patient recruitment and interview scheduling for cognitive debriefing interviews?

- ◯ Not at all capable
- ◯ Partially capable
- ◯ Fully capable
- ◯ Not sure

If you have additional comments about this question, feel free to enter them here:

6. Do you believe that AI is capable of effectively conducting cognitive debriefing interviews with patients?

- ◯ Not at all capable
- ◯ Partially capable
- ◯ Fully capable
- ◯ Not sure

If you have additional comments about this question, feel free to enter them here:

7. Do you believe that AI is capable of improving the process of data review and report preparation for cognitive debriefing interviews?

- ◯ Not at all capable
- ◯ Partially capable
- ◯ Fully capable
- ◯ Not sure

If you have additional comments about this question, feel free to enter them here:

8. Do you believe that AI is capable of improving the eCOA migration and proofreading process?

- Not at all capable
- Partially capable
- Fully capable
- Not sure

If you have additional comments about this question, feel free to enter them here:

9. Overall, do you believe that the introduction of AI into the COA/eCOA translation, linguistic validation, and eCOA migration processes would be a negative or positive development?

- Extremely negative
- Somewhat negative
- Neither negative nor positive
- Somewhat positive
- Extremely positive

If you have additional comments about this question, feel free to enter them here:

10. Do you believe that AI systems involved in COA/eCOA translation, linguistic validation, and eCOA migration processes should be closed systems (i.e., restricted to a company- specific network)?

- Yes
- No
- Not sure

If you have additional comments about this question, feel free to enter them here:

11. Please evaluate whether the COA / eCOA process steps below would be positively or negatively affected by the use of AI, or unaffected by it. If you are not sure how a given process step would be affected, you may skip that row in the table below.

	Positively affected by use of AI	Negatively affected by use of AI	Not affected by use of AI

Creation of Concept Definition/Concept Elaboration document	○	○	○
Dual forward translation	○	○	○
Reconciliation of forward translations	○	○	○
Back-translation	○	○	○
Project Manager review and evaluation of back-translation	○	○	○
Proofreading	○	○	○
Review of clinician review results	○	○	○
Cognitive debriefing interviews with patients	○	○	○
Review of cognitive debriefing interview results	○	○	○
Modification of COA translations for electronic (eCOA) administration	○	○	○
Migration of translated COA text into eCOA technical files	○	○	○
Proofreading of eCOA screen reports	○	○	○
Creation of translation certificate	○	○	○

Other (please identify any missing process steps and evaluate)

12. Please provide any additional comments you have regarding the use of AI within the COA/eCOA translation, linguistic validation, and eCOA migration processes.

13. Is any COA/eCOA process step that could be affected by the use of AI missing from the questions above? Please add any comments about missing steps here.

Pharmaceutical Sponsor Reaction

14. As someone who works for a **pharmaceutical sponsor**, please rate whether you would find COA / eCOA deliverables involving the use of AI acceptable or unacceptable for the following processes:

	Acceptable for use	Unacceptable for use	Not sure
Forward translation process	◯	◯	◯
Back-translation process	◯	◯	◯
Patient recruitment and interview scheduling for cognitive debriefing interviews	◯	◯	◯
Conducting cognitive debriefing interviews with patients	◯	◯	◯
Data review and report preparation for cognitive debriefing interviews	◯	◯	◯
eCOA migration and proofreading	◯	◯	◯

15. For the same processes, if you have additional comments about the acceptability of COA / eCOA deliverables involving the use of AI, please enter them here:

Forward translation process	
Back-translation process	
Patient recruitment and interview scheduling for cognitive debriefing interviews	
Conducting cognitive debriefing interviews with patients	
Data review and report preparation for cognitive debriefing interviews	
eCOA migration and proofreading	

16. Please provide your comments regarding how you, as someone who works for a **pharmaceutical sponsor**, would react to the use of AI in COA translation, back-translation, cognitive debriefing, or eCOA migration processes.

Intellectual Property and Data Confidentiality; Copyright Holder Reaction

17. COA measures frequently contain copyrighted intellectual property. How concerned would you be about the use of AI translation processes having a negative impact on the protection of intellectual property of COA measures?

- ◯ Not at all concerned
- ◯ Somewhat concerned
- ◯ Concerned
- ◯ Extremely concerned

If you have additional comments about this question, feel free to enter them here:

18. How concerned would you be about the use of AI translation processes contributing to inappropriate or illegal use of COA measures from a copyright perspective?

- ◯ Not at all concerned
- ◯ Somewhat concerned
- ◯ Concerned
- ◯ Extremely concerned

If you have additional comments about this question, feel free to enter them here:

19. For the following processes, please rate whether you think **COA copyright holders or developers** would find COA / eCOA deliverables involving the use of AI acceptable or unacceptable:

	Acceptable for use	Unacceptable for use	Not sure
Forward translation process	◯	◯	◯
Back-translation process	◯	◯	◯
Patient recruitment and interview scheduling for cognitive debriefing interviews	◯	◯	◯
Conducting cognitive debriefing interviews with patients	◯	◯	◯
Data review and report preparation for cognitive debriefing interviews	◯	◯	◯
eCOA migration and proofreading			

20. For the same processes, if you have additional comments about the acceptability of COA / eCOA deliverables involving the use of AI from the perspective of **COA copyright holders or developers**, please enter them here:

Forward translation process	
Back-translation process	
Patient recruitment and interview scheduling for cognitive debriefing interviews	
Conducting cognitive debriefing interviews with patients	
Data review and report preparation for cognitive debriefing interviews	
eCOA migration and proofreading	

21. Please provide your comments regarding how you believe **COA copyright holders or developers** would react to the use of AI in COA translation, back-translation, cognitive debriefing, or eCOA migration processes.

Regulatory Reaction

22. For the following processes, please rate whether you think **regulatory bodies** (e.g., FDA, EMA) would find COA / eCOA deliverables involving the use of AI acceptable or unacceptable:

	Acceptable for use	Unacceptable for use	Not sure
Forward translation process	○	○	○
Back-translation process	○	○	○
Patient recruitment and interview scheduling for cognitive debriefing interviews	○	○	○
Conducting cognitive debriefing interviews with patients	○	○	○
Data review and report preparation for cognitive debriefing interviews	○	○	○
eCOA migration and proofreading	○	○	○

23. For the same processes, if you have additional comments about the acceptability of COA / eCOA deliverables involving the use of AI from the perspective of **regulatory bodies** (e.g., FDA, EMA), please enter them here:

Forward translation process	
Back-translation process	
Patient recruitment and interview scheduling for cognitive debriefing interviews	
Conducting cognitive debriefing interviews with patients	
Data review and report preparation for cognitive debriefing interviews	
eCOA migration and proofreading	

24. Please provide your comments regarding how you believe **regulatory bodies** would react to the use of AI in COA translation, back-translation, cognitive debriefing, or eCOA migration processes.

Costs and Timelines

25. How do you think that use of AI during the COA/eCOA translation, linguistic validation, and eCOA migration processes would impact project timelines?

- ◯ Significantly Lengthen
- ◯ Lengthen
- ◯ No impact
- ◯ Reduce
- ◯ Significantly Reduce

If you have additional comments about this question, feel free to enter them here:

26. How do you think that use of AI during the COA/eCOA translation, linguistic validation, and eCOA migration processes would impact project costs?

- ◯ Significantly Increase
- ◯ Increase
- ◯ No impact
- ◯ Decrease
- ◯ Significantly Decrease

If you have additional comments about this question, feel free to enter them here:

27. Please provide your comments on how specifically costs and timelines may be affected by use of AI in COA translation, back-translation, cognitive debriefing, or eCOA migration processes.

28. Thanks for your participation! Please provide the name of the company or organization you represent below.

Thank you very much for completing this survey!

Appendix E : 2005 ISPOR Task Force Members Survey

Introduction

While there has been much discussion around the use of AI for translations in other areas, recommendations pertaining specifically to the use of AI in the context of COA translation and linguistic validation are lacking. The purpose of this survey is to address the lack of consensus around the use of AI in the context of COA translations and linguistic validation and explore the appropriateness and viability of AI use within various stages of COA and eCOA translation, linguistic validation, and electronic (eCOA) migration processes.

For the purposes of this survey, we are using the following definition of AI:

"Artificial intelligence, or AI, is technology that enables computers and machines to simulate human intelligence and problem-solving capabilities."

A more detailed definition of AI, which reinforces the one above, is provided by FDA:

"Artificial Intelligence (AI) is a machine-based system that can, for a given set of human-defined objectives, make predictions, recommendations, or decisions influencing real or virtual environments. AI systems use machine- and human-based inputs to perceive real and virtual environments; abstract such perceptions into models through analysis in an automated manner; and use model inference to formulate options for information or action. AI includes machine learning, which is a set of techniques that can be used to train AI algorithms to improve performance of a task based on data."

Thank you for participating in this survey. Your feedback is appreciated and important. The free text boxes are optional; please complete them only if you wish to. The survey should take about 10-15 minutes, and your feedback is confidential.

AI Capabilities and Appropriateness of Use

1. Do you believe that AI is capable of accurately translating COAs into **languages commonly required** for clinical trials (e.g., French, Spanish, German, Portuguese, Chinese, Japanese, etc.)?

- ◯ Not at all capable
- ◯ Partially capable
- ◯ Fully capable
- ◯ Not sure

If you have additional comments about this question, feel free to enter them here:

2. Do you believe that AI is capable of accurately translating COAs into **all languages that may be required** for use in clinical trials, including those used more rarely (e.g., Zulu, Malayalam, Tagalog, etc.)?

- ◯ Not at all capable
- ◯ Partially capable
- ◯ Fully capable
- ◯ Not sure

If you have additional comments about this question, feel free to enter them here:

3. Do you believe that AI is capable of creating **culturally appropriate** translations of COAs into **languages commonly required** for clinical trials (e.g., French, Spanish, German, Portuguese, Chinese, Japanese, etc.)?

- ◯ Not at all capable
- ◯ Partially capable
- ◯ Fully capable
- ◯ Not sure

If you have additional comments about this question, feel free to enter them here:

4. Do you believe that AI is capable of creating **culturally appropriate** translations of COAs into **all languages that may be required** for use in clinical trials, including those used more rarely (e.g., Zulu, Malayalam, Tagalog, etc.)?

- ◯ Not at all capable
- ◯ Partially capable
- ◯ Fully capable
- ◯ Not sure

If you have additional comments about this question, feel free to enter them here:

5. Do you believe that AI is capable of improving the process of patient recruitment and interview scheduling for cognitive debriefing interviews?

- ◯ Not at all capable
- ◯ Partially capable
- ◯ Fully capable
- ◯ Not sure

If you have additional comments about this question, feel free to enter them here:

6. Do you believe that AI is capable of effectively conducting cognitive debriefing interviews with patients?

- ◯ Not at all capable
- ◯ Partially capable
- ◯ Fully capable
- ◯ Not sure

If you have additional comments about this question, feel free to enter them here:

7. Do you believe that AI is capable of improving the process of data review and report preparation for cognitive debriefing interviews?

- ◯ Not at all capable
- ◯ Partially capable
- ◯ Fully capable
- ◯ Not sure

If you have additional comments about this question, feel free to enter them here:

8. Do you believe that AI is capable of improving the eCOA migration and proofreading process?

- ◯ Not at all capable
- ◯ Partially capable
- ◯ Fully capable
- ◯ Not sure

If you have additional comments about this question, feel free to enter them here:

9. Overall, do you believe that the introduction of AI into the COA/eCOA translation, linguistic validation, and eCOA migration processes would be a negative or positive development?

- ◯ Extremely negative
- ◯ Somewhat negative
- ◯ Neither negative nor positive
- ◯ Somewhat positive
- ◯ Extremely positive

If you have additional comments about this question, feel free to enter them here:

10. Do you believe that AI systems involved in COA/eCOA translation, linguistic validation, and eCOA migration processes should be closed systems (i.e., restricted to a company- specific network)?

- ◯ Yes
- ◯ No
- ◯ Not sure

If you have additional comments about this question, feel free to enter them here:

11. Please evaluate whether the COA / eCOA process steps below would be positively or negatively affected by the use of AI, or unaffected by it. If you are not sure how a given process step would be affected, you may skip that row in the table below.

	Positively affected by use of AI	Negatively affected by use of AI	Not affected by use of AI

Creation of Concept Definition/Concept Elaboration document	○	○	○
Dual forward translation	○	○	○
Reconciliation of forward translations	○	○	○
Back-translation	○	○	○
Project Manager review and evaluation of back-translation	○	○	○
Proofreading	○	○	○
Review of clinician review results	○	○	○
Cognitive debriefing interviews with patients	○	○	○
Review of cognitive debriefing interview results	○	○	○
Modification of COA translations for electronic (eCOA) administration	○	○	○
Migration of translated COA text into eCOA technical files	○	○	○
Proofreading of eCOA screen reports	○	○	○
Creation of translation certificate	○	○	○

Other (please identify any missing process steps and evaluate)

12. Please provide any additional comments you have regarding the use of AI within the COA/eCOA translation, linguistic validation, and eCOA migration processes.

13. Is any COA/eCOA process step that could be affected by the use of AI missing from the questions above? Please add any comments about missing steps here.

ISPOR Guidance Thought Leaders Reaction

14. As a member of the original authorship of the ISPOR Linguistic Validation Best Practices document, please rate whether you would find COA / eCOA deliverables involving the use of AI acceptable or unacceptable for the following processes:

	Acceptable for use	Unacceptable for use	Not sure
Forward translation process	◯	◯	◯
Back-translation process	◯	◯	◯
Patient recruitment and interview scheduling for cognitive debriefing interviews	◯	◯	◯
Conducting cognitive debriefing interviews with patients	◯	◯	◯
Data review and report preparation for cognitive debriefing interviews	◯	◯	◯
eCOA migration and proofreading	◯	◯	◯

15. For the same processes, if you have additional comments about the acceptability of COA / eCOA deliverables involving the use of AI, please enter them here:

Forward translation process	
Back-translation process	
Patient recruitment and interview scheduling for cognitive debriefing interviews	
Conducting cognitive debriefing interviews with patients	
Data review and report preparation for cognitive debriefing interviews	
eCOA migration and proofreading	

16. As a member of the original authorship of the ISPOR Linguistic Validation Best Practices document, please provide your comments regarding how you would react to the use of AI in COA translation, back-translation, cognitive debriefing, or eCOA migration processes.

Intellectual Property and Data Confidentiality; Copyright Holder Reaction

17. COA measures frequently contain copyrighted intellectual property. How concerned would you be about the use of AI translation processes having a negative impact on the protection of intellectual property of COA measures?

- ◯ Not at all concerned
- ◯ Somewhat concerned
- ◯ Concerned
- ◯ Extremely concerned

If you have additional comments about this question, feel free to enter them here:

18. How concerned would you be about the use of AI translation processes contributing to inappropriate or illegal use of COA measures from a copyright perspective?

- ◯ Not at all concerned
- ◯ Somewhat concerned
- ◯ Concerned
- ◯ Extremely concerned

If you have additional comments about this question, feel free to enter them here:

19. For the following processes, please rate whether you think **COA copyright holders or developers** would find COA / eCOA deliverables involving the use of AI acceptable or unacceptable:

	Acceptable for use	Unacceptable for use	Not sure
Forward translation process	◯	◯	◯
Back-translation process	◯	◯	◯
Patient recruitment and interview scheduling for cognitive debriefing interviews	◯	◯	◯
Conducting cognitive debriefing interviews with patients	◯	◯	◯
Data review and report preparation for cognitive debriefing interviews	◯	◯	◯
eCOA migration and proofreading	◯	◯	◯

20. For the same processes, if you have additional comments about the acceptability of COA / eCOA deliverables involving the use of AI from the perspective of **COA copyright holders or developers**, please enter them here:

Forward translation process	
Back-translation process	
Patient recruitment and interview scheduling for cognitive debriefing interviews	
Conducting cognitive debriefing interviews with patients	
Data review and report preparation for cognitive debriefing interviews	
eCOA migration and proofreading	

21. Please provide your comments regarding how you believe **COA copyright holders or developers** would react to the use of AI in COA translation, back-translation, cognitive debriefing, or eCOA migration processes.

Regulatory Reaction

22. For the following processes, please rate whether you think **regulatory bodies** (e.g., FDA, EMA) would find COA / eCOA deliverables involving the use of AI acceptable or unacceptable:

	Acceptable for use	Unacceptable for use	Not sure
Forward translation process	○	○	○
Back-translation process	○	○	○
Patient recruitment and interview scheduling for cognitive debriefing interviews	○	○	○
Conducting cognitive debriefing interviews with patients	○	○	○
Data review and report preparation for cognitive debriefing interviews	○	○	○
eCOA migration and proofreading	○	○	○

23. For the same processes, if you have additional comments about the acceptability of COA / eCOA deliverables involving the use of AI from the perspective of **regulatory bodies** (e.g., FDA, EMA), please enter them here:

Forward translation process	
Back-translation process	
Patient recruitment and interview scheduling for cognitive debriefing interviews	
Conducting cognitive debriefing interviews with patients	
Data review and report preparation for cognitive debriefing interviews	
eCOA migration and proofreading	

24. Please provide your comments regarding how you believe **regulatory bodies** would react to the use of AI in COA translation, back-translation, cognitive debriefing, or eCOA migration processes.

Pharmaceutical Sponsor Reaction

25. For the following processes, please rate whether you think **pharmaceutical sponsors** (COA scientists, HEOR professionals, etc.) would find COA / eCOA deliverables involving the use of AI acceptable or unacceptable:

	Acceptable for use	Unacceptable for use	Not sure
Forward translation process	○	○	○
Back-translation process	○	○	○
Patient recruitment and interview scheduling for cognitive debriefing interviews	○	○	○
Conducting cognitive debriefing interviews with patients	○	○	○
Data review and report preparation for cognitive debriefing interviews	○	○	○
eCOA migration and proofreading	○	○	○

26. For the same processes, if you have additional comments about the acceptability of COA / eCOA deliverables involving the use of AI from the perspective of **pharmaceutical sponsors** (COA scientists, HEOR professionals, etc.), please enter them here:

Forward translation process	
Back-translation process	
Patient recruitment and interview scheduling for cognitive debriefing interviews	
Conducting cognitive debriefing interviews with patients	
Data review and report preparation for cognitive debriefing interviews	
eCOA migration and proofreading	

27. Please provide your comments regarding how you believe **pharmaceutical sponsors** would react to the use of AI in COA translation, back-translation, cognitive debriefing, or eCOA migration processes.

Costs and Timelines

28. How do you think that use of AI during the COA/eCOA translation, linguistic validation, and eCOA migration processes would impact project timelines?

- ◯ Significantly Lengthen
- ◯ Lengthen
- ◯ No impact
- ◯ Reduce
- ◯ Significantly Reduce

If you have additional comments about this question, feel free to enter them here:

29. How do you think that use of AI during the COA/eCOA translation, linguistic validation, and eCOA migration processes would impact project costs?

- ◯ Significantly Increase
- ◯ Increase
- ◯ No impact
- ◯ Decrease
- ◯ Significantly Decrease

If you have additional comments about this question, feel free to enter them here:

30. Please provide your comments on how specifically costs and timelines may be affected by use of AI in COA translation, back-translation, cognitive debriefing, or eCOA migration processes.

31. Thanks for your participation! Please provide the name of the company or organization you represent below.

Thank you very much for completing this survey!
